# A rapid and affordable screening platform for membrane protein trafficking

**DOI:** 10.1186/s12915-015-0216-3

**Published:** 2015-12-17

**Authors:** Joshua C. Snyder, Thomas F. Pack, Lauren K. Rochelle, Subhasish K. Chakraborty, Ming Zhang, Andrew W. Eaton, Yushi Bai, Lauren A. Ernst, Larry S. Barak, Alan S. Waggoner, Marc G. Caron

**Affiliations:** Departments of Cell Biology, Duke University Medical Center, Durham, NC 27710 USA; Departments of Medicine and Neurobiology, Duke University Medical Center, Durham, NC 27710 USA; Department of Pharmacology and Cancer Biology, Duke University Medical Center, Durham, NC 27710 USA; Molecular Biosensor and Imaging Center, Carnegie Mellon University, Pittsburgh, PA 15213 USA; Department of Biology, Molecular Biosensor and Imaging Center, Carnegie Mellon University, Pittsburgh, PA 15213 USA

**Keywords:** G protein-coupled receptor, High-throughput screening, Lgr5, Receptor trafficking

## Abstract

**Background:**

Membrane proteins regulate a diversity of physiological processes and are the most successful class of targets in drug discovery. However, the number of targets adequately explored in chemical space and the limited resources available for screening are significant problems shared by drug-discovery centers and small laboratories. Therefore, a low-cost and universally applicable screen for membrane protein trafficking was developed.

**Results:**

This high-throughput screen (HTS), termed IRFAP-HTS, utilizes the recently described MarsCy1-fluorogen activating protein and the near-infrared and membrane impermeant fluorogen SCi1. The cell surface expression of MarsCy1 epitope-tagged receptors can be visualized by simple addition of SCi1. User-friendly, rapid, and quantitative detection occurs on a standard infrared western-blotting scanner. The reliability and robustness of IRFAP-HTS was validated by confirming human vasopressin-2 receptor and dopamine receptor-2 trafficking in response to agonist or antagonist. The IRFAP-HTS screen was deployed against the leucine-rich G protein-coupled receptor-5 (Lgr5). Lgr5 is expressed in stem cells, modulates Wnt/ß-catenin signaling, and is therefore a promising drug target. However, small molecule modulators have yet to be reported. The constitutive internalization of Lgr5 appears to be one primary mode through which its function is regulated. Therefore, IRFAP-HTS was utilized to screen 11,258 FDA-approved and drug-like small molecules for those that antagonize Lgr5 internalization. Glucocorticoids were found to potently increase Lgr5 expression at the plasma membrane.

**Conclusion:**

The IRFAP-HTS platform provides a versatile solution for screening more targets with fewer resources. Using only a standard western-blotting scanner, we were able to screen 5,000 compounds per hour in a robust and quantitative assay. Multi-purposing standardly available laboratory equipment eliminates the need for idiosyncratic and more expensive high-content imaging systems. The modular and user-friendly IRFAP-HTS is a significant departure from current screening platforms. Small laboratories will have unprecedented access to a robust and reliable screening platform and will no longer be limited by the esoteric nature of assay development, data acquisition, and post-screening analysis. The discovery of glucocorticoids as modulators for Lgr5 trafficking confirms that IRFAP-HTS can accelerate drug-discovery and drug-repurposing for even the most obscure targets.

**Electronic supplementary material:**

The online version of this article (doi:10.1186/s12915-015-0216-3) contains supplementary material, which is available to authorized users.

## Background

The advent of high-throughput screening (HTS) has enabled successful unbiased drug-discovery and fostered the development of novel therapies [1]. Arguably the most fruitful targets in HTS platforms have been membrane proteins, which comprise 22 % of the proteins encoded by the genome and are targeted by 60 % of the approved drugs available today. Incredibly, almost half of these drugs are directed at the rhodopsin-like class I G protein-coupled receptor (GPCR) superfamily [2]. Many of these receptors have underlying roles in a myriad of diseases, including cancer, heart disease, diabetes, and mental illness. Therefore, membrane proteins represent a gold mine of targets that must be screened in order to fully exploit their rich therapeutic potential.

For instance, the expression of the leucine-rich G protein-coupled receptor-5 (Lgr5) was recently shown to identify stem cells of the intestine [3]. More recent evidence has demonstrated that adult tissue-specific stem cells of the stomach [4], hair follicle [5], and mammary gland [6, 7], can be identified solely through expression of Lgr5. The Lgr5-expressing stem cell is a critical contributor to tissue maintenance, and may also be the cell of origin in gastrointestinal cancers [8, 9]. Lgr5 is a GPCR whose biochemical and cellular properties have evaded investigators since its discovery in 1998 [10, 11]. Therefore, Lgr5 is an exciting membrane protein target for which small molecule modulators are unfortunately lacking. Previously, we have shown that Lgr5 constitutively internalizes from the plasma membrane and retrograde traffics to the trans-Golgi network [12]. Inhibiting this internalization resulted in the formation of ‘cytonemes’, which are ultra-long actin-rich signaling filopodia capable of scaffolding cell signaling at a distance [13, 14]. Together, these data suggest that internalization and trafficking of Lgr5 may be critical for fine-tuning its function. Therefore, small molecule modulators of Lgr5 trafficking may prove to be a powerful strategy for pharmacologically modulating stem cell activity.

High-throughput screening platforms for plasma membrane receptors have had success due to reliable cell-based systems for monitoring a diversity of downstream messengers [15], such as cAMP, Ca^2+^ mobilization, and Rho GTPase activation, or translocation of adaptor molecules after activation such as β-arrestin [16]. However, in most cases, these assays are highly idiosyncratic and consequently require specialized protocol development. HTS becomes especially challenging for those receptors that are biologically rich but have non-canonical signaling or remain uncharacterized, such as Lgr5. Whereas receptor signaling is specialized, all classes of plasma membrane receptors are synthesized in the endoplasmic reticulum, before their targeting to the cell surface. Thus, plasma membrane trafficking is the single universal feature of membrane receptor protein regulation. Reasons that HTS trafficking screens are not more often utilized include a lack of reagent universality, expensive imaging equipment, and confounding background fluorescence that in many instances requires sophisticated de-convolution algorithms to identify subpopulations of membrane proteins.

Our solution relies upon a new class of genetically encoded fluorogen activating proteins (FAPs) that immuno-react with and induce the fluorescence of spectrally tunable and weakly fluorescent compounds (fluorogens) [17]. The synthesis of a novel near-infrared FAP (MarsCy1):Fluorogen (SCi1) and its versatile use for in vivo imaging of tumors was recently described [18]. Herein, we pair MarsCy1 with a low cost infrared (IR) western-blotting scanner for simultaneous multi-plate analysis of receptor trafficking in a high-throughput system that we term IRFAP-HTS. Fluorogen fluorescence is increased up to 20,000-fold when receptors genetically fused to the FAP bind to the fluorogen. Real-time monitoring of FAP-tagged membrane protein trafficking occurs by simple addition of fluorogen without the need for reagent washes or highly-automated equipment [17, 19]. We validated IRFAP-HTS with GPCRs of well-known pharmacology and then confirmed its utility by screening and identifying small molecule modulators of Lgr5 trafficking.

## Results

### Validating a sensor for visualizing cell surface expression of membrane proteins

**Fig. 1:**
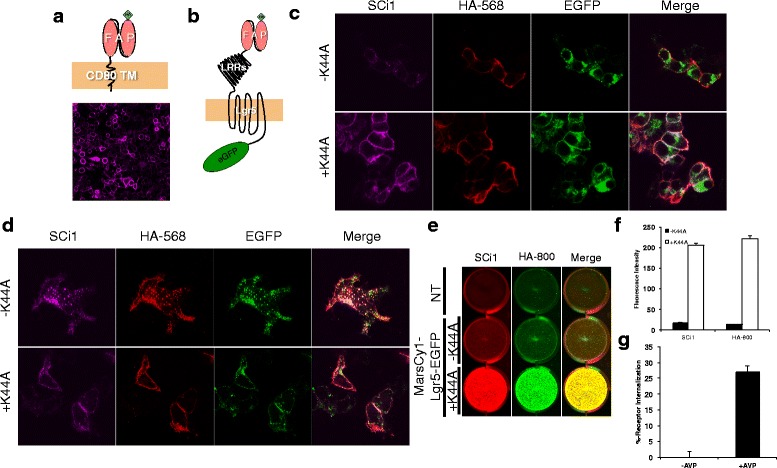
Quantitative scanning of MarsCy1-tagged membrane proteins in a modular plate-based format. **a** Top: Cartoon depicting HA-FAP fused to the N-terminus of the CD80 transmembrane domain. Bottom: Confocal imaging of FAP-CD80 transfected cells stained with SCi1. **b** Cartoon depicting HA-FAP fused to the N-terminus of Lgr5 and EGFP fused to the C-terminus. **c** Confocal imaging of FAP-tagged Lgr5-EGFP transfected cells that were co-labeled with SCi1 (magenta) and a primary HA-epitope antibody (Red, secondary retrieval – 568 nm) at 4 °C to block receptor internalization and were chased in (**d**) for 30 minutes at 37 °C to allow constitutive internalization of Lgr5 (EGFP, green) (K44A, A dominant-negative Dynamin I mutant that inhibits receptor endocytosis when over-expressed). **e** Infrared plate imaging of a 12-well plate (see Additional file 4: Figure S4 for entire plate and time-course) with FAP-tagged Lgr5 pulsed with SCi1 and a primary HA-epitope antibody at 4 °C and then fixed. Non-permeabilized cells were scanned on the plate at 700 nm (red, SCi1) and 800 nm (green, HA secondary retrieval-800 nm) (NT: non-transfected). **f** Integrated fluorescence intensity from (**e**). **g** MarsCy1-tagged hV2R was transiently transfected in HEK cells on a 24-well plate and stimulated with vehicle (–AVP) or the V2R ligand AVP [10 μM] for 1 hour. Cells were SCi1 stained, scanned, and quantified

Quantifying receptor expression using affordable multi-purpose equipment available in most labs would be particularly advantageous to small academic laboratories. The excitation and emission of the MarsCy1/SCi1 pair (Ex/Em: 703 nm/733 nm) occurs in a range suitable for use on many IR-western blotting scanners and is easily resolved from the visible spectrum, which is often filled by other reporters (i.e. EGFP and RFP). The feasibility of the system was first tested by fusing an N-terminal Hemagglutinin (HA)-MarsCy1-FAP to Lgr5-EGFP (MarsCy1-Lgr5-EGFP). The membrane permeant near-IR fluorogen (SC1) stained MarsCy1-Lgr5-EGFP and verified the expected localization of Lgr5 in intracellular vesicles (Additional file 1: Figure S1). The membrane impermeant variant of SC1 (SCi1) was next tested for its ability to confer cell-surface labeling of membrane proteins. SCi1 enabled robust cell surface labeling of a MarsCy1-CD80 transmembrane fused protein, with undetectable intracellular staining (Fig. 1a).

The ability for SCi1 to label cell surface MarsCy1-tagged receptors and permit real-time imaging of trafficking was evaluated and directly compared to standard HA-immunolabeling. Cells expressing MarsCy1-Lgr5-EGFP were co-pulsed with HA-antibody and SCi1 at 4 °C to label the cell-surface pool of the receptor (Fig. 1b–d and Additional file 2: Figure S2). In the absence of chase, cells displayed low level Lgr5 surface expression, as revealed by equivalent SCi1 and HA-immunoreactivity. This corresponds with the previously described intracellular localization of Lgr5 and is again revealed by native EGFP fluorescence (Fig. 1c). Previous findings demonstrate that Lgr5 constitutive internalization can be blocked by overexpression of a dominant negative form of Dynamin I (K44A) [12]. K44A expression robustly increased cell surface staining by SCi1 and HA-antibody staining (Fig. 1c). When cells were chased for 30 minutes at 37 °C, the cell surface fraction of Lgr5 rapidly internalized into vesicles (Fig. 1d). K44A significantly blunted this internalization (Fig. 1d). Importantly, MarsCy1 fusion did not perturb normal Lgr5 trafficking (Additional file 3: Figure S3 and Additional file 4: Figure S4) and could be used in combination with SCi1 to monitor the subcellular distribution of Lgr5 and its internalization by confocal microscopy (Fig. 1).

Previously, we quantified receptor internalization by HA-immunolabeling cells in a plate-format and scanning on an IR western blotting scanner [12]. Therefore, we directly compared HA-immunolabeling and MarsCy1:SCi1 staining on cells expressing MarsCy1-Lgr5-EGFP that were co-pulsed with SCi1 and HA-antibody (800 nm). The entire 12-well plate was scanned on a LiCOR-Odyssey® IR imaging system and quantified. MarsCy1-Lgr5-EGFP cells weakly expressed Lgr5 on the plasma membrane, whereas those cells co-transfected with K44A demonstrated a robust increase in Lgr5 cell surface expression. SCi1 and HA-800 perform similarly in this assay and quantitatively confirm the confocal imaging data analysis (Fig. 1e,f and Additional file 4: Figure S4). We also generated a MarsCy1-fused human vasopressin receptor-2 (V2R) and demonstrated that IR-scanning of MarsCy1:SCi1 enabled quantification of prototypical agonist-induced GPCR internalization (Fig. 1g). At steady-state, the V2R exhibits very little internalization [20]. MarsCy1 staining and live confocal imaging confirmed this finding, verified that SCi1 is membrane impermeant, and demonstrated that MarsCy1-tagging can be used to visualize trafficking in living cells in real-time (Additional file 5: Movie S1). Collectively, these data demonstrated that MarsCy1-tagged GPCRs and the membrane impermeant fluorogen SCi1 can be used to quantifiably and reliably assess surface expression of GPCRs.

### Cell surface rescue of GPCRs

**Fig. 2:**
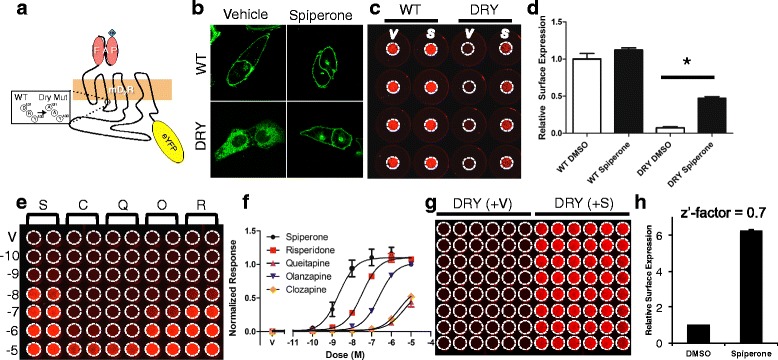
Monitoring cell surface rescue of a mutant intracellularly mis-localized membrane protein. **a** Cartoon depicting HA-FAP fused to the N-terminus of wild-type (WT) D2R or DRY-AAY (DRY) D2R and EYFP on the C-terminus. **b** Confocal imaging for EYFP-tagged WT-D2R or DRY-D2R treated with vehicle (DMSO) or the D2R antagonist spiperone [10 μM]. **c** Infrared plate imaging for membrane impermeable SCi1 bound to FAP-tagged WT- or DRY-D2R treated with DMSO or spiperone (S, 10 μM) overnight. **d** Quantification of triplicate experiments represented in panel (**c**) and normalized relative to WT-D2R surface expression (* denotes significant difference by ANOVA and post hoc Tukey-analysis). **e** Small-scale dose-response screen of overnight treatment with small molecule antagonists using quantitative infrared plate imaging (V, DMSO Vehicle; S, Spiperone; R, Risperidone; Q, Quetiapine; O, Olanzapine; C, Clozapine. 10-10 (-10) M to 10-5 (-5) M). **f** Quantification of triplicate experiments of the representative image in (**e**) and normalized to spiperone (S). **g** Infrared image of 10 μM spiperone DRY-D2R surface expression rescue for Z’-factor analysis. **h ** Quantification of panel (**g**) and calculation of a Z’-factor (* denotes significant difference by student’s unpaired *t*-test)

For MarsCy1:SCi1 to be a valid screening platform, the assay must enable the robust quantification of receptor cell-surface expression in a high-throughput multi-well plate format. Since small molecule modulators of Lgr5 function have not been reported, we used a prototypical GPCR with better characterized trafficking and signaling modes to validate this system. Most class 1 GPCRs possess a canonical ‘DRY’ motif at the end of transmembrane-domain three which can stabilize the receptor in an inactive state and is also important in G protein-coupling (Fig. 2a) [21, 22]. Mutations to this domain contribute to disease by resulting in constitutive activation and internalization of the receptor [20, 23] in addition to misfolded receptor that accumulates in the ER [24]. Therefore, we tested if the MarsCy1:SCi1 system could be used to identify small molecules that rescue surface expression of a mutant GPCR with a known pharmacological profile. We generated an N-terminally MarsCy1-tagged wild-type human D2 dopamine receptor and a DRY to AAY mutant D2R (DRY). Inclusion of an EYFP tag at the D2R C-terminus, revealed by confocal microscopy that wild-type human D2 dopamine receptor displayed normal cell surface expression relative to the internalized and intracellularly localized DRY mutant. As expected, overnight treatment with the D2R antagonist spiperone significantly rescued surface expression of the DRY mutant (Fig. 2b).

SCi1 staining enabled robust visualization and quantification of plasma membrane surface rescue for the DRY mutant receptor (Fig. 2c, d). This assay was then scaled up to a 96-well plate format for screening MarsCy1 DRY mutant expressing cells against six characterized D2R antagonists. As expected, each antagonist was able to partially rescue DRY mutant surface expression (Fig. 2e, f). Z’-factor analysis provides a statistical measure of HTS robustness and reliability with values >0.5 considered to be an excellent screening platform [25]. A Z’-factor of 0.7 was calculated for spiperone-mediated DRY surface expression in a 96-well plate format. These data demonstrated that the MarsCy1:SCi1 system can report on the cell surface expression of GPCRs in a format amenable for HTS.

### HTS for candidate small molecules that rescue Lgr5 surface expression

**Fig. 3:**
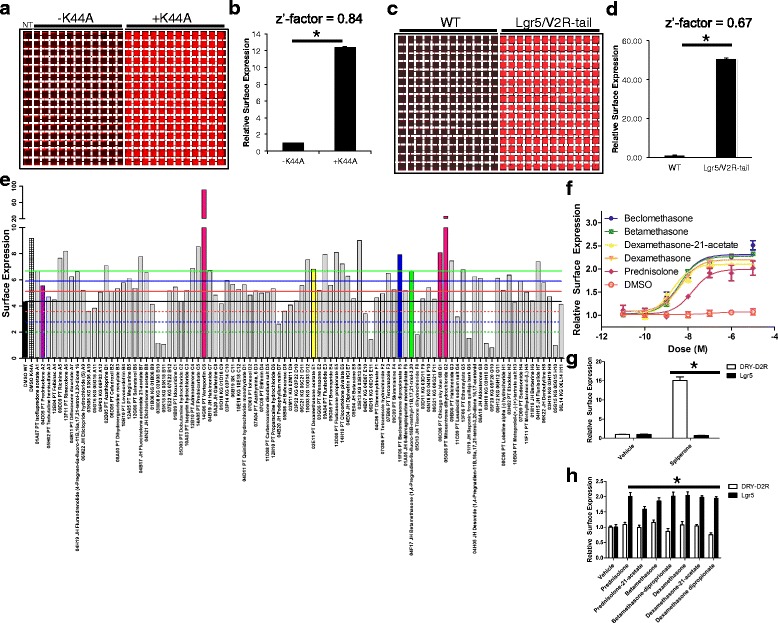
Screening for small molecule modulators of Lgr5 surface expression. **a** Infrared image of a stable U2OS cell line expressing MarsCy1-Lgr5 in a 384-well plate without (–K44A) and with transient transfection (+K44A) of Dynamin I K44A (NT, parental U2OS cells). **b** Quantification of (**a**) and Z’-factor analysis. **c** Infrared image of a stable U2OS cell lines expressing MarsCy1-Lgr5 compared to MarsCy1-Lgr5/V2R-tail (NT, parental U2OS cells). **d** Quantification of (c) and Z’-factor analysis. **e** A total of 91 hits were cherry picked and incubated overnight at 37 °C on stable MarsCy1-Lgr5-EGFP cells. Black bar, wild-type Lgr5; Hatched bar, +K44A control; Pink bar, autofluorescent compounds (violet, yellow, blue, and green bars as in 3f). Hits were measured against the ± 1, 2, 3 standard deviations from wild-type DMSO mean (green, blue, and red lines). Each compound is described according to plate ID, library name (JH, John’s Hopkins; PT, Prestwick; KG, Kinase Gold), common drug name, and position on the secondary screening plate. **f** Synthetic glucocorticoid receptor agonists from (**e**) were purchased, in addition to dexamethasone, and screened in a dose-response assay. **g** and **h** Spiperone and glucocorticoids, respectively, increase plasma membrane expression of D2R-DRY and Lgr5

The MarsCy1:SCi1 system was then tested for its utility as a screening platform. Lgr5 was chosen as a prime candidate due to its unique expression in stem cells, its well-documented constitutive internalization properties [12, 26], and its lack of reported small molecule modulators. Before the Lgr5 screen was initiated, the MarsCy1:SCi1 system was first reformatted and tested in a 384-well system to increase throughput to 5,000 wells/hour. Z’-factors for the assay were calculated with either K44A overexpression or comparison to a cell line expressing an Lg5/V2R fusion previously shown to have increased surface expression (MarsCy1-Lgr5/V2R-EGFP) [12]. Both comparisons demonstrated that the 384-well format provides an excellent screening platform that we have termed IRFAP-HTS (Z’-factor = 0.84 and Z’-factor = 0.67) (Fig. 3a-d). A total of 11,258 compounds from five libraries of diverse chemical space were screened using IRFAP-HTS. These libraries target kinases, GPCRs, and FDA-approved clinically effective drugs and included John’s Hopkins Clinical Compound Library (1,518 compounds, 60 hits, 4.0 % hit-rate, Additional file 6: File S1) [27], Kinase Gold Library (3,519 compounds, 29 hits, 0.8 % hit-rate, Additional file 7: File S2), Prestwick Library (1,120 compounds, 129 hits, 11.5 % hit-rate, Additional file 8: File S3), SigmaKinase (101 compounds, 1 hit, 0.9 % hit-rate, Additional file 9: File S4), and ActProb5k (5,000 compounds, 51 hits, 1.0 % hit rate, Additional file 10: File S5).

We found 270 total hits that increased Lgr5 surface expression, equating to a primary screen hit rate of 2.4 %. We cherry-picked 91 of these hits and performed a secondary screen at 20 μM and 0.2 μM and found that 25 % of these were reproducible at 20 μM (Fig. 3e and Additional file 11: Figure S5). Some hits were also discounted due to their fluorescent properties overlapping with the same spectrum as the IR detector. Among the most promising hits, we were intrigued by the discovery of several glucocorticoids, some of which were hits across multiple libraries. Each of the glucocorticoids potently and significantly increased Lgr5 surface expression (Fig. 3f). Other hits, while reproducible, were either beyond the scope of this study or have limited availability for further testing outside of in-house synthesis. The specificity of glucocorticoids was determined in a counter-screen against the D2R receptor using MarsCy1-DRY expressing cells. The membrane permeant antagonist spiperone robustly rescues DRY surface expression but has no effect on Lgr5 (Fig. 3g). In contrast, glucocorticoids increase Lgr5 surface expression while displaying no activity toward D2R DRY (Fig. 3h and Table 1). We tested dexamethasone for direct binding to Lgr5. Dexamethasone did not bind to Lgr5 with high-affinity (Additional file 12: Figure S6). This implies that Lgr5 surface expression might be indirectly regulated by glucocorticoid receptor signaling. These data demonstrate that the IRFAP-HTS is a user-friendly, modular, and rapidly deployable screening platform.

**Table 1:** Small molecules

Drug	CAS No.	MW	Supplier	Cat#	Solvent	Stock
DEX	50-02-2	392.46	Sigma	D1756	100 % EtOH	10 mM
DEX dipropionate	55541-30-5	504.59	TimTec	ST024761	100 % EtOH	10 mM
DEX 21-acetate	1177-87-3	434.5	Sigma	D1881	100 % EtOH	10 mM
Beclomethasone dipropionate	9/8/5534	521.04	Sigma	B3022	100 % EtOH	10 mM
Betamethasone	378-44-9	392.46	TRC	B327000	100 % EtOH	10 mM
Betamethasone 17,21-dipropionate	5593-20-4	504.59	Sigma	B1152	100 % EtOH	10 mM
Prednisolone	50-24-8	360.44	Sigma	P6004	100 % EtOH	10 mM
Prednisolone 21-acetate	52-21-1	402.48	Sigma	P8650	100 % EtOH	10 mM

## Discussion

Historically, and as a result of reliance on state-of-the-art instrument technologies, screening platforms have required significant upfront capital investment in robotics, assay-specific detection systems, and workspace [28]. The IRFAP-HTS platform described in this study instead enables real-time HTS of membrane protein surface expression in an easy-to-use, versatile, and rapidly deployable format. The success of our platform lies in the ability to robustly and precisely screen for membrane protein trafficking with limited upfront investment. Overall, this is a substantial departure from standard HTS platforms. In summary, the IRFAP-HTS provides a system where a single investigator in a small laboratory space can easily and affordably screen their chosen target against modestly sized compound libraries (10,000 compounds) in a single week.

A combination of several desirable features enabled realization of this technology. In contrast to other HTS platforms, the FAP-based screen only requires access to standard cell culture facilities, low-cost manual pipetting systems, and a highly-versatile IR western-blotting scanner commonly available to most basic science labs. In this study, we were able to utilize 12-, 24-, 96-, 384-, and single-well formats with little to no change in experimental workflow. This enables acquisition of close to 5,000 data points in 1 hour, since six 384-well plates can be processed in parallel within approximately 30 minutes. The images generated in our assay encompass the plate in its entirety and enables immediate and visually qualitative hit-analysis. This mitigates the need for extensive data analysis, de-convolution, and software investment. Our assay has essentially no, to at most, very low background due to the spectral characteristics of the near-IR region in cell-based systems. MarsCy1-tagged protein targets are easily generated using common molecular biological techniques and in the multitude of proteins tested have not significantly impacted normal trafficking. The specificity of MarsCy1-induced SCi1 fluorescence is due to the very high-affinity antibody-based fluorescence enhancement. This chemistry enables scanning within minutes of SCi1 addition without the need for washing unbound material. This obviates the need for additional liquid handling and plate-washers and thereby reduces cell loss that can accompany extensive handling. In addition, MarsCy1-SCi1 enables co-screening with other fluorescent assay reporters, such as those far removed from the IR spectrum (Additional file 13: Figure S7).

Proof-of-principle for the IRFAP-HTS was achieved by focusing on three different applications for membrane proteins. This included agonist-induced internalization of the V2R, antagonist drug rescue of the mutant D2R, and drug discovery for the small-molecule-orphan Lgr5. These data demonstrated that the IRFAP-assay can be utilized in agonist or antagonist mode without any modifications to the platform. For HTS, we chose Lgr5 as a prototype since we are interested in its unique trafficking properties, its role in stem cell biology and cancer, and its history as a difficult target to study. The proposed Lgr5 ligands, Rspondin [29–31] and Norrin [32], do not activate classical G protein or ß-arrestin dependent signaling pathways, making standard screening assays difficult. This is despite the conservation of key signaling determinants for typical GPCR signal transduction and a fully functional ß-arrestin translocation domain [33]. Surprisingly, the IRFAP-HTS system identified synthetic glucocorticoid receptor (GR) agonists as potent modulators of Lgr5 trafficking.

Glucocorticoids exert many effects on tissue development and maturation. Work from Florence Moog’s lab in the 1950s demonstrated that glucocorticoids drive proper maturation of the intestinal epithelium [34, 35]. Moog’s work enabled a series of investigations that further outlined roles for glucocorticoids in the maturation of the intestine. These studies confirmed Moog’s original observations, identified the window of glucocorticoid responsiveness, and first suggested that cells within the crypt might be the primary target in the epithelium [36–40]. Interestingly, GR agonists act on intestinal epithelial cells to inhibit proliferation, promote morphological changes, and restructure the trans-Golgi network (TGN) [41]. Therefore, in the context of our previous findings [12], the GR-mediated restructuring of the TGN network may provide one mechanism whereby the TGN-localized Lgr5 can more efficiently traffic to the plasma membrane. These data all point to an unexpected but important link between GRs and Lgr5. The clinical and therapeutic implications of this finding will be the subject of future research.

Repurposing drugs is a viable alternative to deep high-throughput screening of millions of combinatorial compounds with unknown pharmacological profiles [27]. In fact, directed screens against FDA-approved compounds have been successful in defining new activity for many commonly used and clinically relevant compounds [42]. Many of these hits can be used as a scaffold for chemical evolution to further refine their activity profile and efficacy [43]. Therefore, using IRFAP-HTS, small academic laboratories can now institute drug-repurposing programs targeted against their membrane protein of interest.

## Conclusions

A major objective of the post-genomic era has been to identify molecular targets of disease and develop new modes of pharmacological intervention. Therefore, we have developed a versatile IRFAP-HTS for membrane protein trafficking so that any lab can easily screen approximately 10,000 small molecules per week and have a high-probability of finding a significant hit. This study demonstrates the viability of this system by using NIH-procured libraries and affordably priced commercial libraries. Using IRFAP-HTS, even the smallest of laboratories can make a minimal investment of $5,000 to obtain and screen approximately 1,500 FDA-approved compounds [27] against any user-defined membrane protein. We expect that the IRFAP-HTS will transform drug-discovery into an open-source pursuit within reach of all basic science and clinical research laboratories and, for the first time, provide a platform for the synergistic exploration of disease targets in the public domain.

## Methods

### Biological constructs

The described MarsCy1 sequence [18] was PCR-amplified and overlap-exchanged into the N-terminus of a human Lgr5 C-terminal EGFP fusion previously described. To confer appropriate trafficking MarsCy1 was inserted immediately following the 5’-signal sequence of Lgr5. Human D2R and V2R lack cleavable signal sequences due to the absence of a large extracellular N-terminal domain. Therefore, to confer correct ER transport and plasma membrane insertion for each receptor, the signal sequence of Lgr5 was included on the MarsCy1-tagged D2R and V2R. A MarsCy1-CD80 fusion is described in Zhang et al. [18].

### MarsCy1 staining

SC1 and SCi1 were synthesized according to Zhang et al. [18] and reconstituted in ethanol with 5 % acetic acid at 200 μM or 78 μM, respectively. Live or fixed cells were incubated with 200 nM SC1 or 20 nM SCi1 for 5 minutes and then imaged. No washing was performed unless indicated.

### Antibody staining

Live cells were pulse-labeled with HA-antibody (1:500 dilution in staining media) as previously described. Following a 45-minute pulse on ice, cells were washed and then fixed or chased. A secondary Donkey-anti-Mouse-800 nm (LiCOR®, cat#926-32212, Lincoln, NE) or Goat-anti-Mouse-568 (LifeTechnologies, cat# A-11004, Carlsbad, CA) was used for IR scanning or confocal microscopy, respectively.

### MarsCy1 confocal imaging

Confocal imaging for SC1 and EGFP was performed on a Zeiss LSM510 confocal microscope (Carl Zeiss Microscopy, Germany). For three-color imaging of SCi1, EGFP, and HA immunolabeling (568 nm) a Zeiss LSM780 confocal microscope was used (Carl Zeiss Microscopy, Germany).

### MarsCy1 IR scanning

A LI-COR Odyssey® (LI-COR Biosciences, Lincoln, NE) system was used for scanning MarsCy1 tagged receptors stained with SCi1 or SC1 in the 700 nm channel. Focal offsets were adjusted accordingly to match the focal plane of the plate. For cells stained with SCi1 and immunolabeled for HA (800 nm), plates were scanned at 700 nm and 800 nm, respectively.

### IRFAP-HTS protocols

A detailed description of all HTS protocols, the primary data generated, mining algorithms for processing hits, and the library descriptions can be found as Microsoft Excel worksheets (Microsoft Office 2011, Microsoft Corporation, Redman, WA) in Additional file 6: File S1, Additional file 7: File S2, Additional file 8: File S3, Additional file 9: File S4 and Additional file 10: File S5. Briefly, MarsCy1-tagged cell lines were incubated with drugs overnight in 384-well plates, and then fixed and stained with SCi1, or stained with SCi1 and scanned live, as indicated. Up to six-plates were scanned at 700 nm using a LI-COR Odyssey® in approximately 30 minutes. Data were exported and merged with the library key files (available at https://web.duke.edu/gpcr-assay/) in Microsoft Access (Microsoft Office 2011, Microsoft Corporation, Redman, WA). Merged files were then mined in Excel.

### Chemical cataloguing

Top hits were purchased and freshly made for further evaluation. These include those listed in Table 1.

### Statistical analysis

Statistical analysis was performed in GraphPad Prism (GraphPad Software, Inc., La Jolla, CA).

## Additional files

Additional file 1: Figure S1. Testing MarsCy1-Lgr5 expression and activity toward SC1. (a) Live HEK cells stained with SC (30 min at 0. 2 μM) and imaged at 488 and 633 nm with a confocal microscope. No staining was observed. (b–d) HEK cells were transiently transfected with MarsCy1-Lgr5 and imaged at 488 and 633 nm by confocal microscopy. (b) SC unstained control. (c) Live HEK cells stained with SC (0. 2 μM) for 30 minutes without a washout. (d) Live HEK cells stained with SC (0. 2 μM) for 30 minutes, washed, fixed, and imaged. (e) LI-COR imaging of non-transfected or MarsCy1-Lgr5 transfected cells that were stained with SC with or without washing. The integrated intensity is also shown. (f) U2OS clones stably expressing MarsCy1-Lgr5 (duplicates (e/f)) compared to parenteral cells display varying degrees of MarsCy1 induced SC activity. Average integrated intensity is shown. (PDF 1545 kb) Additional file 2: Figure S2. Single color controls for multi-color confocal microscopy. MarsCy1-Lgr5-EGFP was stained with SCi1 and HA-568 or unstained (EGFP). Spectral gating and laser power was set on a LSM-780 to eliminate channel overlap. (PDF 5943 kb) Additional file 3: Figure S3. MarsCy1-tagged Lgr5 retains normal trafficking behavior. HEKT cells were transiently transfected with MarsCy1-Lgr5-EGFP. (a) Confocal images of cells pulse labeled with HA-antibody (568) for 45 minutes on ice and chased for 0, 30, or 120 minutes. (Top: 3xHA-Lgr5-EGFP vs Bottom: Mars1-Lgr5-EGFP). (b) Same as (a) but with overexpression of the endocytosis inhibitor dynamin K44A. (Red: 568 nm/HA; Green: 488 nm/EGFP; Blue: 633 nm/Nuclear Dye. (PDF 20474 kb) Additional file 4: Figure S4. MarsCy1-tagged Lgr5 retains normal trafficking behavior and can be quantified on an infrared western blotting imager. HEKT cells were transiently transfected with MarsCy1-tagged Lgr5-EGFP on a 12-well plate. Cells were co-pulsed with SCi1 and HA-antibody on ice for 45 minutes and then chased for (a) 0, (b) 30, or (c) 120 minutes and scanned on a LiCOR Odyssey for SCi1 (700 nm) and HA (800 nm). (d) Quantification of (a–c). (PDF 4232 kb) Additional file 5: Movie S1. MarsCy1-V2R live imaging. MarsCy1 tagged V2R was transiently expressed in HEK cells. SCi1 was added to cell culture media. Cell membranes were fluorescent within 1 minute and reached a peak at approximately 3.5 minutes, at which point the time-lapse began. Images were acquired every 30 seconds for 15 minutes. Note that internalization of unstimulated V2R is not detected and that SCi1 does not penetrate the plasma membrane. No washing was performed since unbound SCi1 is only weakly fluorescent. (MOV 451 kb) Additional file 6: File S1. John’s Hopkins Clinical Compound Library. Lgr5 IRFAP-HTS results provided in a Microsoft Excel Spreadsheet with three worksheets that include (Description) the experimental overview, (Data) Raw and Analyzed data, (PivotTable) and a pivot table for data mining and determination of hits. (XLS 1334 kb) Additional file 7: File S2. Kinase Gold Library. Lgr5 IRFAP-HTS results provided in a Microsoft Excel Spreadsheet with three worksheets that include: (Description) the experimental overview, (Data) Raw and Analyzed data, (PivotTable) and a pivot table for data mining and determination of hits. (XLS 2999 kb) Additional file 8: File S3. Prestwick Library. Lgr5 IRFAP-HTS results provided in a Microsoft Excel Spreadsheet with three worksheets that include: (Description) the experimental overview, (Data) Raw and Analyzed data, (PivotTable) and a pivot table for data mining and determination of hits. (XLSX 344 kb) Additional file 9: File S4. Sigma Kinase Library. Lgr5 IRFAP-HTS results provided in a Microsoft Excel Spreadsheet with three worksheets that include: (Description) the experimental overview, (Data) Raw and Analyzed data, (PivotTable) and a pivot table for data mining and determination of hits. (XLS 218 kb) Additional file 10: File S5. ActProb5K Library. Lgr5 IRFAP-HTS results provided in a Microsoft Excel Spreadsheet with three worksheets that include: (Description) the experimental overview, (Data) Raw and Analyzed data, (PivotTable) and a pivot table for data mining and determination of hits. (XLSX 2266 kb) Additional file 11: Figure S5. Rescreening of hits at 0.2 μM. Hits were individually selected and incubated overnight at 0.2 μM overnight on stable MarsCy1-Lgr5-EGFP cells in a 384-well plate. Black, WT Lgr5; Hatched bar, +K44A control; Pink bar, reportedly autofluorescent compounds; violet, yellow, blue, and green bars correspond to Fig. 1f tested GR agonists. JH, John’s Hopkins; PT, Preswick; KG, Kinase Gold. Each compound is described according to plate ID, library name, common drug name, and position on the secondary screening plate. KG lacks common names. (PDF 1088 kb) Additional file 12: Figure S6. Dexamethasone does not directly bind Lgr5. Membrane proteins were extracted from U2OS cells stably expressing WT Lgr5, Lgr5/V2r, or control cells expressing human neurotensin-2 (NTR2). (h + c) Lgr5 extracts were incubated with 10 μM dexamethasone (c: cold) washed and then incubated with ^3^H-dexamethasone (h: hot). (hot) Lgr5 extracts were incubated with 10 μM ^3^H-dexamethasone. As a control NTR2 binding experiments were similarly performed with hot 10 nM ^3^H-neurotensin or cold neurotensin. As expected, pre-incubation with cold neurotensin reduces binding of hot ligand. Specific binding of dexamethasone was not observed for Lgr5 or in the negative control NTR2. (PDF 40 kb) Additional file 13: Figure S7. Multi-color HTS of Lgr5 membrane expression β-arrestin-2 translocation. Stable U2OS cells expressing MarsCy1-Lgr5-V2R and β-arrestin-2-EGFP were screened against the ActivProb library (plate 1 shown) and fixed with 4 % PFA. (a) Cells were stained for SCi1 and imaged on an IR-western blotting scanner (700 nm). (Column 1: U2OS parenteral line, Columns 2, 23, and 24: DMSO, Columns 3–22: Drug). (b) The plate was then imaged at 257.6× magnification on a Zeiss AxioZoom Microscope. Eight images for each well were imaged using automated ZenBlue software totaling 2,880 images in 20 minutes, as previously published and termed ArrestinZoom. (Column 1 was not imaged as it does not have GFP expression). (c) Box in (b) shown at 35 % magnification and (d) asterisk denoted area in (c) shown at 100 %. No hits were identified but these data demonstrate the ease with dual-function screening can be performed. (PDF 1328 kb)

## Abbreviations

FAPs: Fluorogen activating proteins
GPCR: G protein-coupled receptor
GR: Glucocorticoid receptor
HA: Hemagglutinin
HTS: High-throughput screening
IR: Infrared
K44A: Dominant negative form of Dynamin I
Lgr5: Leucine-rich G protein-coupled receptor-5
TGN: Trans-Golgi network
V2R: Vasopressin receptor-2
