# Supplementary figures and images for: A rapid and affordable screening platform for membrane protein trafficking
Source: BMC Biol. 2015 Dec 17;13:107. doi: 10.1186/s12915-015-0216-3 (PMC4683952; doi:10.1186/s12915-015-0216-3)

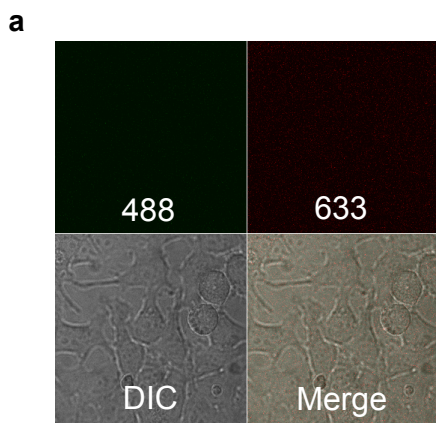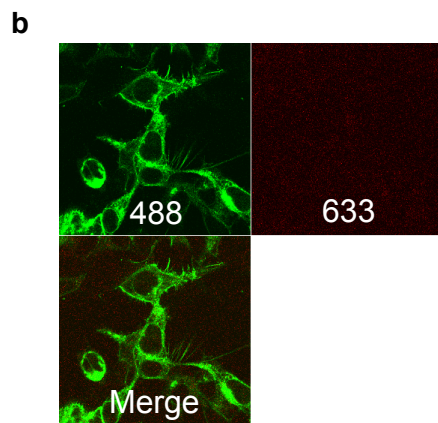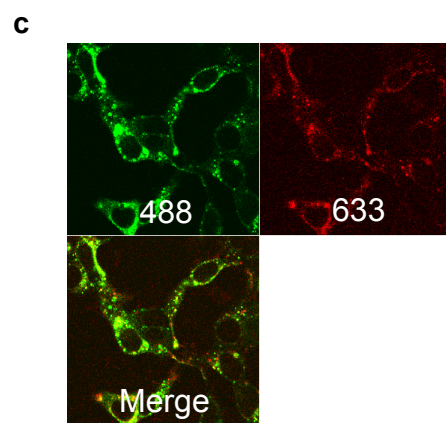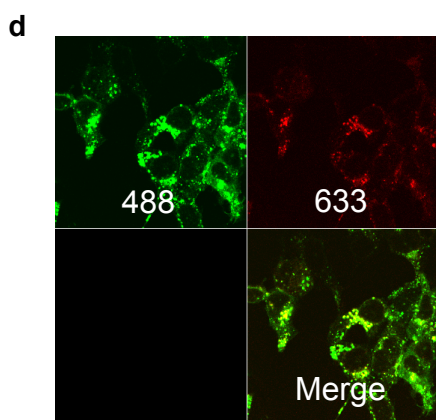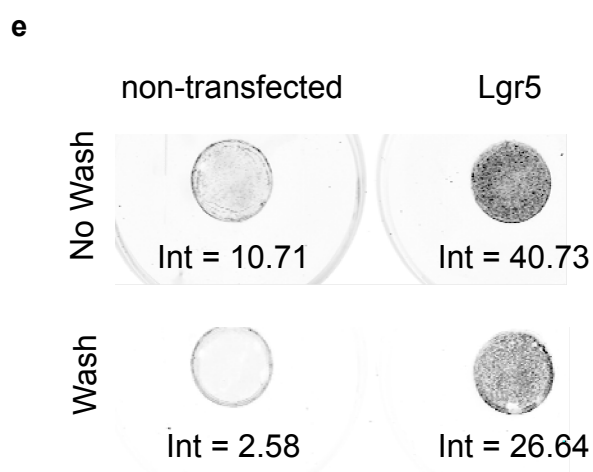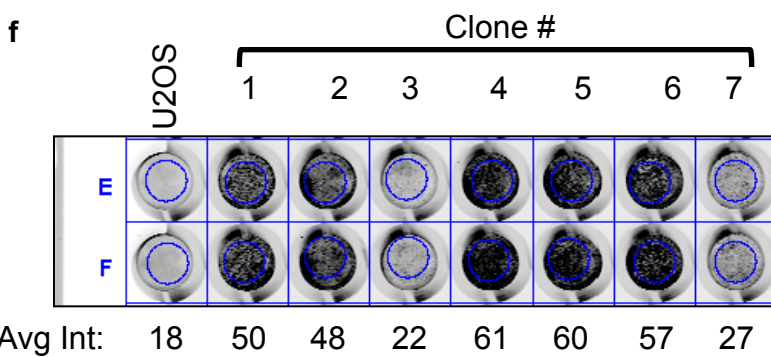

Supplement: Additional file 1: Figure S1. — Testing MarsCy1-Lgr5 expression and activity toward SC1. (a) Live HEK cells stained with SC (30 min at 0. 2 μM) and imaged at 488 and 633 nm with a confocal microscope. No staining was observed. (b–d) HEK cells were transiently transfected with MarsCy1-Lgr5 and imaged at 488 and 633 nm by confocal microscopy. (b) SC unstained control. (c) Live HEK cells stained with SC (0. 2 μM) for 30 minutes without a washout. (d) Live HEK cells stained with SC (0. 2 μM) for 30 minutes, washed, fixed, and imaged. (e) LI-COR imaging of non-transfected or MarsCy1-Lgr5 transfected cells that were stained with SC with or without washing. The integrated intensity is also shown. (f) U2OS clones stably expressing MarsCy1-Lgr5 (duplicates (e/f)) compared to parenteral cells display varying degrees of MarsCy1 induced SC activity. Average integrated intensity is shown. (PDF 1545 kb) [file 12915_2015_216_MOESM1_ESM.pdf]

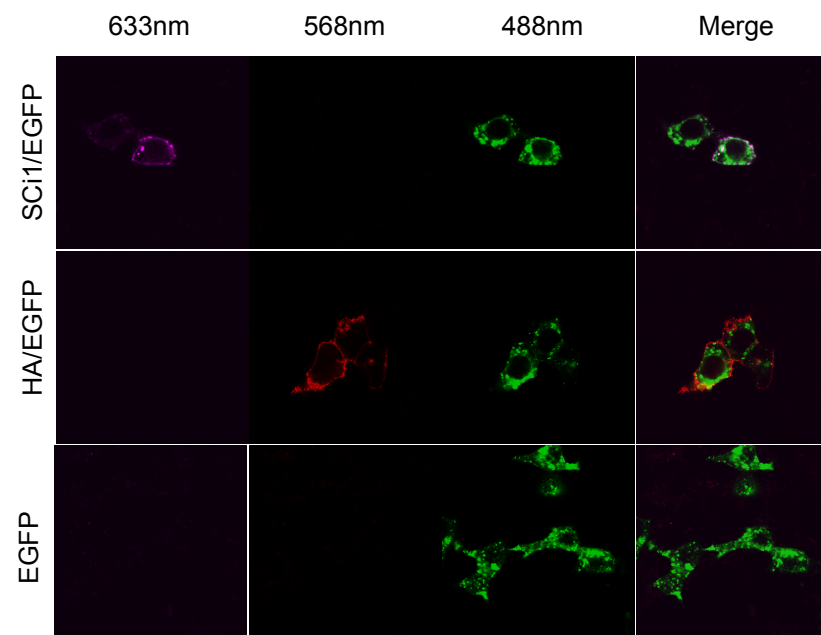

Supplement: Additional file 2: Figure S2. — Single color controls for multi-color confocal microscopy. MarsCy1-Lgr5-EGFP was stained with SCi1 and HA-568 or unstained (EGFP). Spectral gating and laser power was set on a LSM-780 to eliminate channel overlap. (PDF 5943 kb) [file 12915_2015_216_MOESM2_ESM.pdf]

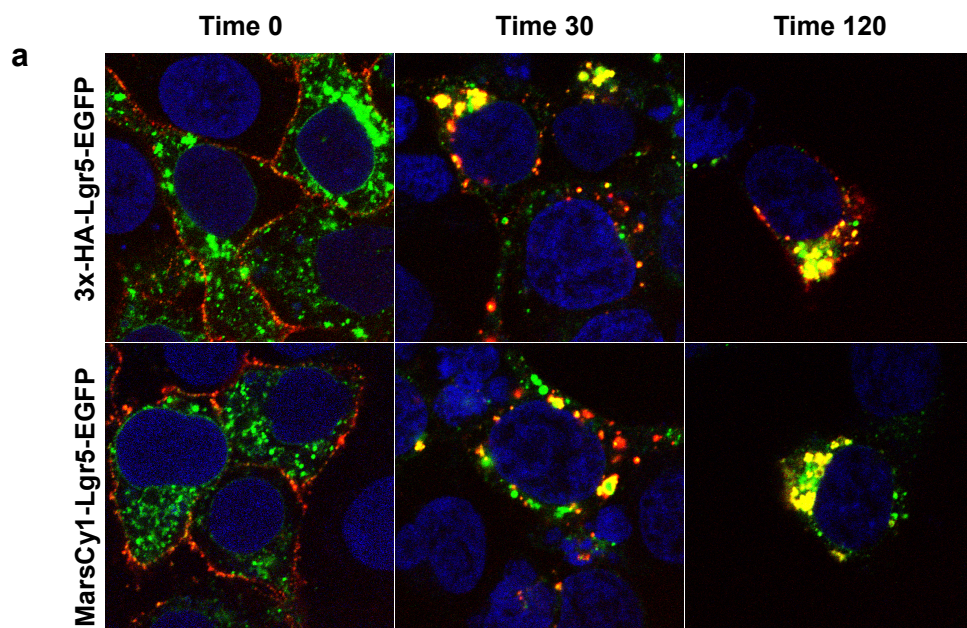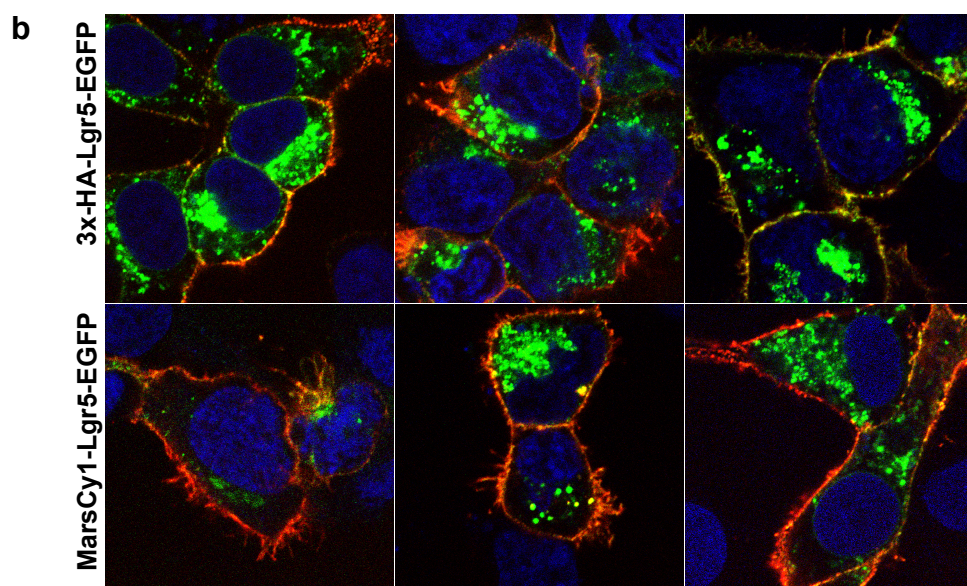

Supplement: Additional file 3: Figure S3. — MarsCy1-tagged Lgr5 retains normal trafficking behavior. HEKT cells were transiently transfected with MarsCy1-Lgr5-EGFP. (a) Confocal images of cells pulse labeled with HA-antibody (568) for 45 minutes on ice and chased for 0, 30, or 120 minutes. (Top: 3xHA-Lgr5-EGFP vs Bottom: Mars1-Lgr5-EGFP). (b) Same as (a) but with overexpression of the endocytosis inhibitor dynamin K44A. (Red: 568 nm/HA; Green: 488 nm/EGFP; Blue: 633 nm/Nuclear Dye. (PDF 20474 kb) [file 12915_2015_216_MOESM3_ESM.pdf]

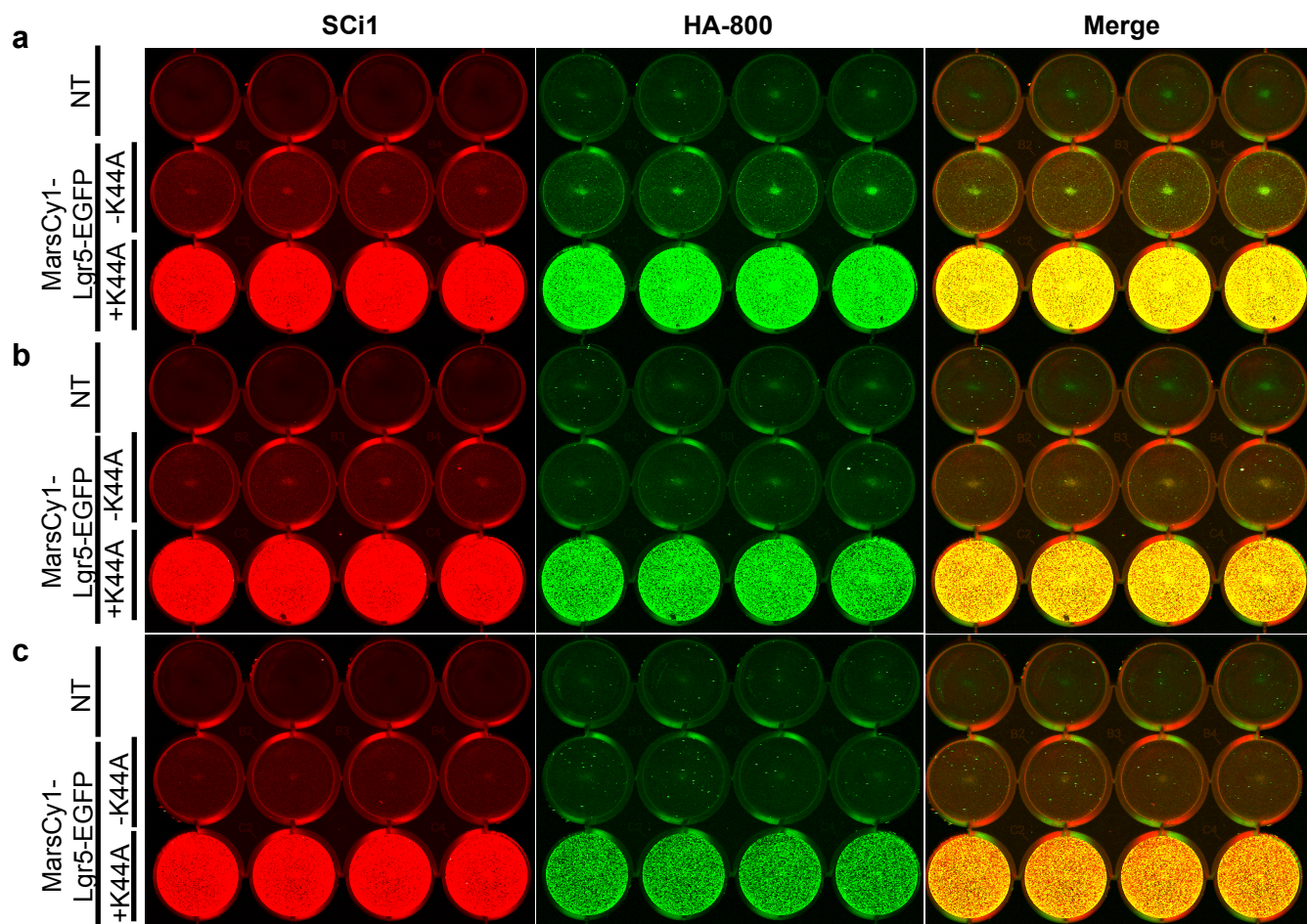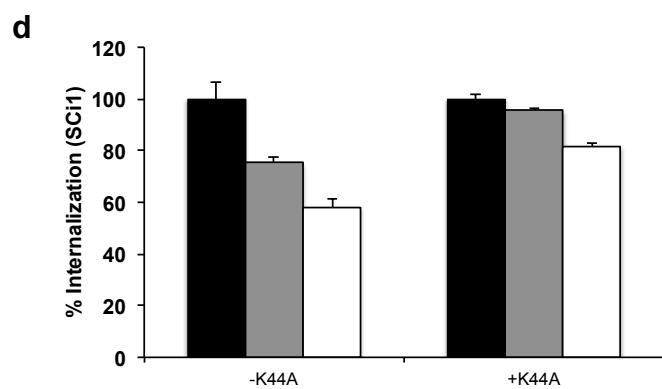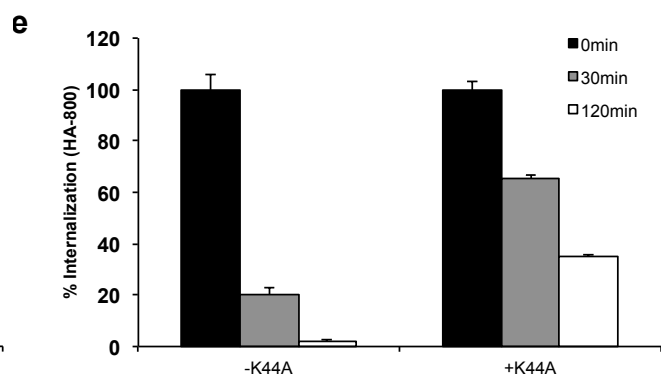

Supplement: Additional file 4: Figure S4. — MarsCy1-tagged Lgr5 retains normal trafficking behavior and can be quantified on an infrared western blotting imager. HEKT cells were transiently transfected with MarsCy1-tagged Lgr5-EGFP on a 12-well plate. Cells were co-pulsed with SCi1 and HA-antibody on ice for 45 minutes and then chased for (a) 0, (b) 30, or (c) 120 minutes and scanned on a LiCOR Odyssey for SCi1 (700 nm) and HA (800 nm). (d) Quantification of (a–c). (PDF 4232 kb) [file 12915_2015_216_MOESM4_ESM.pdf]

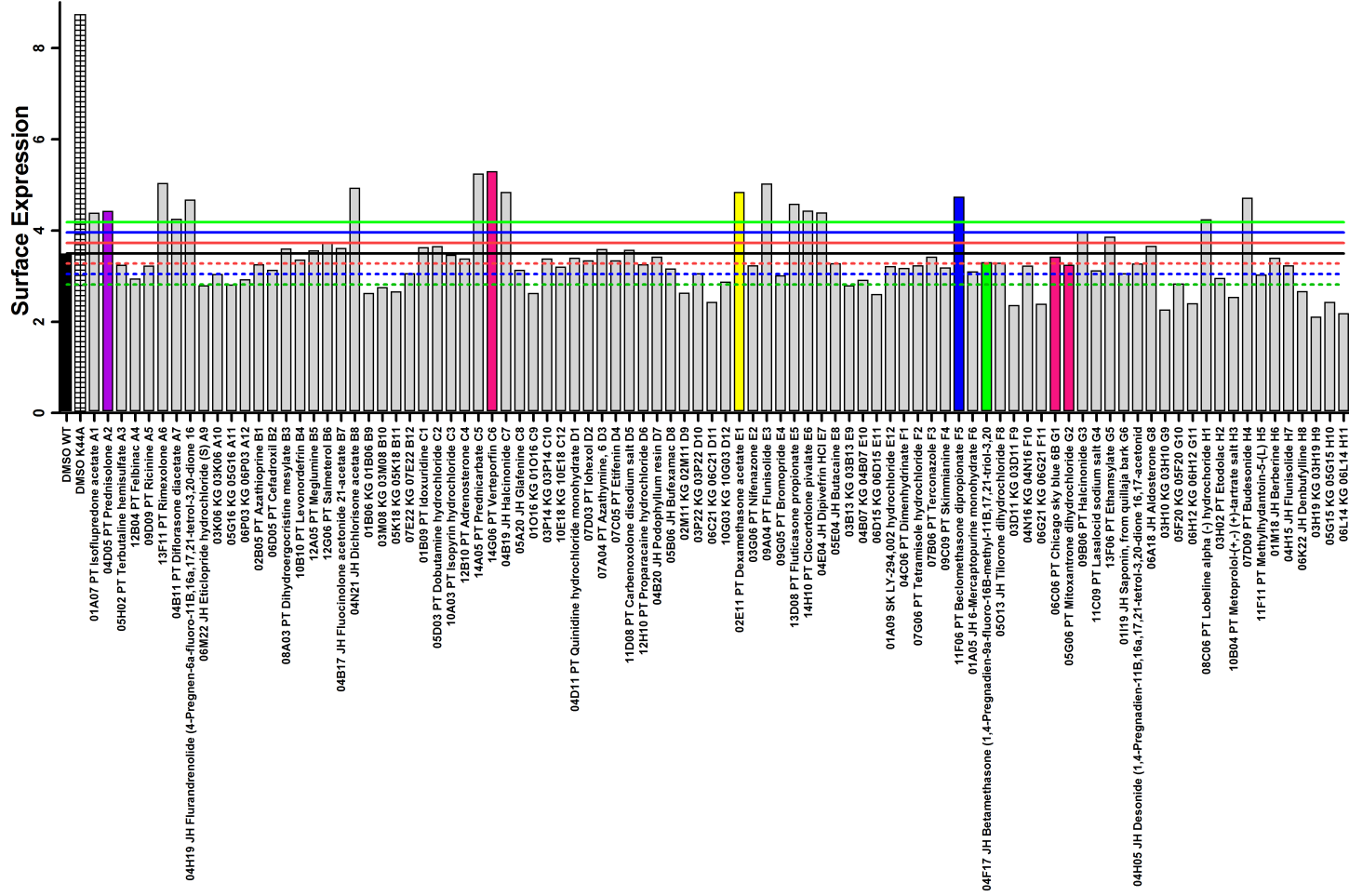

Supplement: Additional file 11: Figure S5. — Rescreening of hits at 0.2 μM. Hits were individually selected and incubated overnight at 0.2 μM overnight on stable MarsCy1-Lgr5-EGFP cells in a 384-well plate. Black, WT Lgr5; Hatched bar, +K44A control; Pink bar, reportedly autofluorescent compounds; violet, yellow, blue, and green bars correspond to Fig. 1f tested GR agonists. JH, John’s Hopkins; PT, Preswick; KG, Kinase Gold. Each compound is described according to plate ID, library name, common drug name, and position on the secondary screening plate. KG lacks common names. (PDF 1088 kb) [file 12915_2015_216_MOESM11_ESM.pdf]

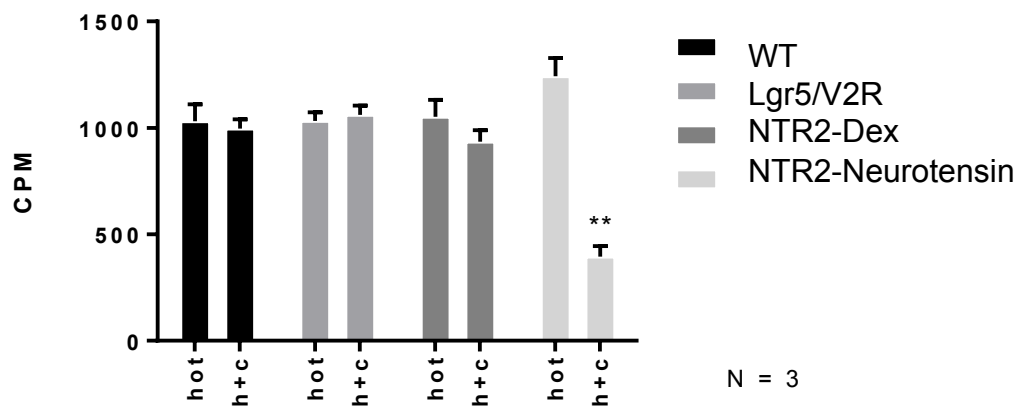

Supplement: Additional file 12: Figure S6. — Dexamethasone does not directly bind Lgr5. Membrane proteins were extracted from U2OS cells stably expressing WT Lgr5, Lgr5/V2r, or control cells expressing human neurotensin-2 (NTR2). (h + c) Lgr5 extracts were incubated with 10 μM dexamethasone (c: cold) washed and then incubated with 3H-dexamethasone (h: hot). (hot) Lgr5 extracts were incubated with 10 μM 3H-dexamethasone. As a control NTR2 binding experiments were similarly performed with hot 10 nM 3H-neurotensin or cold neurotensin. As expected, pre-incubation with cold neurotensin reduces binding of hot ligand. Specific binding of dexamethasone was not observed for Lgr5 or in the negative control NTR2. (PDF 40 kb) [file 12915_2015_216_MOESM12_ESM.pdf]

**a**

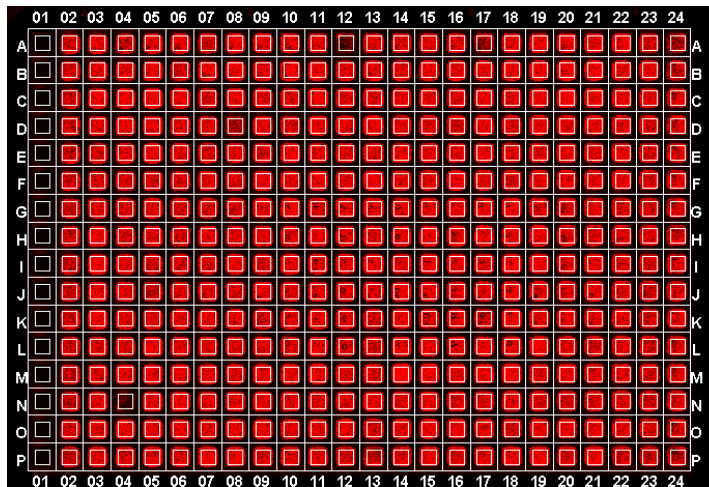

**b**

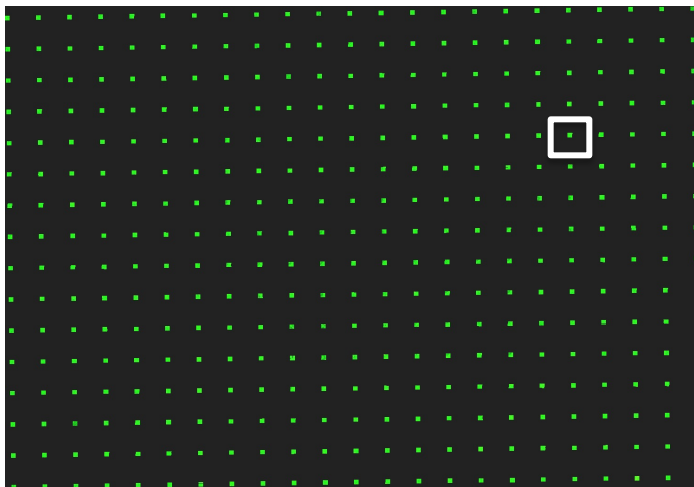

**c**

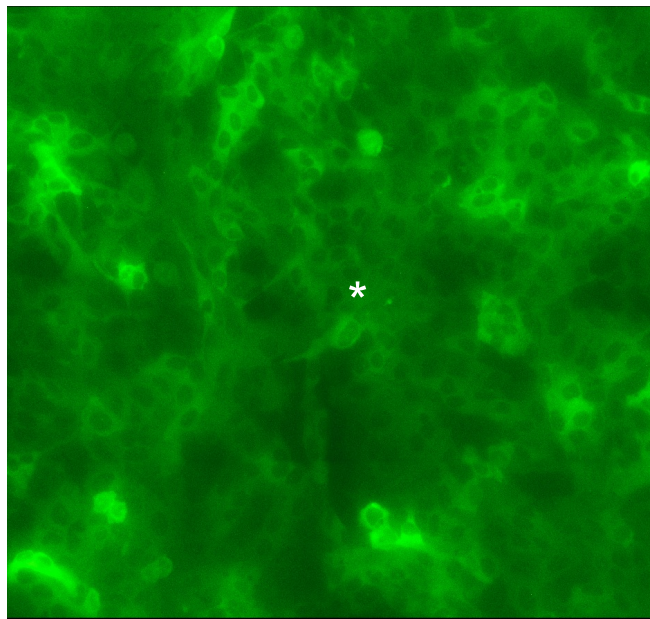

**d**

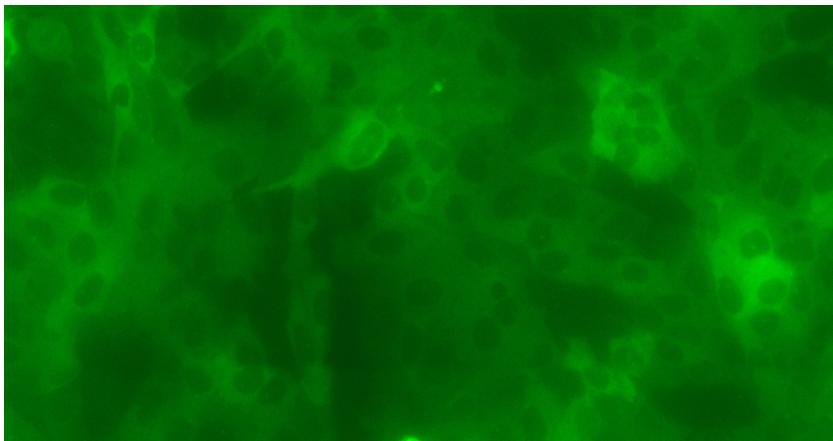

Supplement: Additional file 13: Figure S7. — Multi-color HTS of Lgr5 membrane expression β-arrestin-2 translocation. Stable U2OS cells expressing MarsCy1-Lgr5-V2R and β-arrestin-2-EGFP were screened against the ActivProb library (plate 1 shown) and fixed with 4 % PFA. (a) Cells were stained for SCi1 and imaged on an IR-western blotting scanner (700 nm). (Column 1: U2OS parenteral line, Columns 2, 23, and 24: DMSO, Columns 3–22: Drug). (b) The plate was then imaged at 257.6× magnification on a Zeiss AxioZoom Microscope. Eight images for each well were imaged using automated ZenBlue software totaling 2,880 images in 20 minutes, as previously published and termed ArrestinZoom. (Column 1 was not imaged as it does not have GFP expression). (c) Box in (b) shown at 35 % magnification and (d) asterisk denoted area in (c) shown at 100 %. No hits were identified but these data demonstrate the ease with dual-function screening can be performed. (PDF 1328 kb) [file 12915_2015_216_MOESM13_ESM.pdf]
